# Different Language Modalities Yet Similar Cognitive Processes in Arithmetic Fact Retrieval

**DOI:** 10.3390/brainsci12020145

**Published:** 2022-01-22

**Authors:** Ilaria Berteletti, Sarah E. Kimbley, SaraBeth J. Sullivan, Lorna C. Quandt, Makoto Miyakoshi

**Affiliations:** 1 Ph.D. in Educational Neuroscience Program, Gallaudet University, Washington, DC 20002, USA; sarah.kimbley@gallaudet.edu (S.E.K.); sarabeth.sullivan@gallaudet.edu (S.J.S.); lorna.quandt@gallaudet.edu (L.C.Q.); 2 Swartz Center for Computational Neuroscience, Institute for Neural Computation, University of California, San Diego, CA 92093, USA; mmiyakoshi@ucsd.edu

**Keywords:** event-related potentials, arithmetic facts, deaf native signers, language modality, problem size effect, operation type effect, American Sign Language

## Abstract

Does experience with signed language impact the neurocognitive processes recruited by adults solving arithmetic problems? We used event-related potentials (ERPs) to identify the components that are modulated by operation type and problem size in Deaf American Sign Language (ASL) native signers and in hearing English-speaking participants. Participants were presented with single-digit subtraction and multiplication problems in a delayed verification task. Problem size was manipulated in small and large problems with an additional extra-large subtraction condition to equate the overall magnitude of large multiplication problems. Results show comparable behavioral results and similar ERP dissociations across groups. First, an early operation type effect is observed around 200 ms post-problem onset, suggesting that both groups have a similar attentional differentiation for processing subtraction and multiplication problems. Second, for the posterior-occipital component between 240 ms and 300 ms, subtraction problems show a similar modulation with problem size in both groups, suggesting that only subtraction problems recruit quantity-related processes. Control analyses exclude possible perceptual and cross-operation magnitude-related effects. These results are the first evidence that the two operation types rely on distinct cognitive processes within the ASL native signing population and that they are equivalent to those observed in the English-speaking population.

## 1. Introduction

Studies in arithmetical cognition have been focusing on how experience, training and teaching practices can impact the neurocognitive substrates supporting arithmetic processing [1]. So far, studies have shown real-time learning effects [2,3,4,5], focused strategies training effects [2,5] and even cultural-linguistic differences [6] to determine the extent to which the neural substrates as well as the cognitive strategies used in arithmetic can be and are malleable. A question that has been overlooked is whether language modality, signed instead of spoken, may impact the neural network and strategies involved in single-digit arithmetic processing.

Sign languages, such as American Sign Language (ASL), are complete natural languages that have the same linguistic properties and complexity as spoken languages: they possess grammatical rules, syntax and phonological and morphological properties [7]. Signed languages are perceived visually and expressed manually; thus, information is conveyed through substantially different sensory modalities. Given that our brains and cognitive processes are shaped by experience [8], it is no surprise that using sign language, and not deafness, has been shown to enhance or modify both cognitive and neural processes [9,10,11,12,13,14,15,16]. Research has also shown that sign languages, acquired early, rely on a network including the same left-lateralized brain areas supporting spoken languages [13,14,15,16,17,18]. These studies have been central in demonstrating how the neural substrates for language processing are function-dependent and modality-independent [17]. However, much less is known about the impact of language modality on the neurocognitive processes supporting proficient arithmetic processing. Here, we aim to investigate whether early and lifelong exposure to a visuo-manual language (i.e., a signed language) combined with early profound to severe hearing loss may modify the cognitive processes and neural response involved in solving arithmetic problems.

So far, the literature in cognitive and neuroscience of arithmetic has shown that different arithmetic operations are mostly solved through different strategies that rely on partially distinct brain networks [19,20,21,22]. Electrophysiological studies support this distinction as well [23,24,25]. Subtraction problems rely on procedures and quantity manipulation, whereas multiplication problems rely on the phonological loop and verbal retrieval of stored facts [26,27]. Solving subtraction problems relies on bilateral parietal and posterior areas typically involved in quantity processing (intraparietal sulcus, IPS) and visuospatial manipulation (posterior superior parietal lobule, PSPL) [23,26,28]. Solving multiplication problems, on the other hand, activates a left-lateralized network in the temporal (superior and middle temporal gyri, STG and MTG) and inferior frontal cortices (inferior frontal gyrus, IFG) commonly activated in phonological processing [24,26]. This differentiation of brain networks appears to be experience-driven, where practice and training increase the observed neural differentiation [3,4,5,28,29,30]. Additionally, problem size has been shown to modulate the reliance on the different networks, with larger problems being more likely solved through computation-based strategies [26,31,32,33,34]. An open question is whether the differentiation between operation types is related to language modality and if current findings are therefore specific to the spoken language.

By comparing the electrophysiological correlates evoked by multiplication and subtraction problems in hearing English speaking and Deaf native ASL signing participants, we will be able to test whether modality has an impact on the brain functions involved and, if so, at which stage these come into play. We chose the event-related potential approach as a first investigation comparing the strategies used by Deaf native signers and English-speaking participants. As Hinault and Lemair [35] explain, the EEG method (i.e., either the ERP or ERSP) can provide electrophysiological signatures in support of different arithmetic strategies and show that these strategies are implemented differently depending on problem size and operation type. Here, we will leverage the strength of the EEG method to investigate whether the Deaf native signers and English-speaking participants show distinct electrophysiological signatures and if these differ depending on the operation type as well as problem size. We use a delayed verification paradigm in which participants are presented with small and large single-digit subtraction and multiplication problems as well as extra-large subtraction problems. These two operation types have shown the greatest dissociation in strategies and neural correlates [21,22]. We also added an extra-large subtraction condition to equate the overall numerical magnitude of large single-digit multiplication problems. In our English-speaking control group, we expect to see differences related to operation type in the earlier time windows (i.e., components). Prior studies have suggested that the early modulations are indicative of differential allocation of attentional resources supporting distinct cognitive strategies [23,24,25,36]. Only one study identified differences as early as 100 ms post-problem onset [37], whereas most other studies found differences beyond 200 ms and up to 300 ms following problem onset [23,24,25]. Because prior research has shown that Deaf native signers recruit similar left-lateralized language areas for language processing, and if these language-based processes are also uniquely supporting multiplication-specific retrieval [26,28], we expect to find a similar early dissociation for the Deaf signing group. Alternatively, research has also shown that processing a sign language might recruit more bilateral frontotemporal brain areas as well as parietal areas supporting visuospatial processes [13]. These additional processes related to sign language could influence the neuro-cognitive processes involved in solving arithmetic problems. Therefore, it is possible that our Deaf native signing group either shows no distinction between operations or shows a dissociation between the operations at a different timepoint. If a lifelong exposure to a signed language impacts how Deaf signers allocate resources and attentional processes in relation to the two operation types, we would expect an early interaction between group and operation type where only our hearing participants show a dissociation as observed in prior studies. In relation to problem size, prior work has shown modulation over a late component indicating greater use of computational instead of retrieval strategies [30,32,33,37]. This modulation is expected to be stronger for subtraction problems as these have shown to rely on computation [23,26,28]. Furthermore, the modulation of the amplitudes on the later component will have to be consistent with the three different levels of problem size for subtraction problems. If our Deaf native signers were to rely on retrieval for both problem types, we might not see a modulation with problem size for subtraction problems at this later component.

## 2. Materials and Methods

### 2.1. Participants

Participants were recruited through a mix of university and local advertising strategies. An initial screening was performed by email and eligible participants were then invited to the lab. To be included in the study, participants had to be between 18 and 35 years of age, either be native English speakers with no hearing loss or native ASL signers (exposure before age 2) identifying as Deaf and having used ASL during their formative education (i.e., School for the Deaf, Deaf Program in mainstream schools or interpreter support in mainstream schools). After removing participants due to technical failures (missing files, equipment failure, recording issues, *n* = 9) and participants with low accuracy (below 70%: *n* = 3 English speakers and *n* = 2 Deaf ASL Signers), 29 ASL native signers and 35 English speakers were included in the analyses. The mean age for the ASL native group was 23.7 years (SD = 6.10) and for the English-speaking group was 27.2 (SD = 8.06). A *t*-test revealed that age was not significantly different between groups (*p* > 0.1). Gender distribution was 15 females and 14 males in the ASL native group and 24 females and 10 males, with one participant declining to disclose the information for the English-speaking group. A chi-square distribution showed that gender across groups was not significantly different from random ($\chi^{2}\;$ = 2.36, *p* > 0.1). All but one English-speaking participant were right-handed.

### 2.2. Procedure

While participants were being fitted with the EEG cap, they signed the consent form, responded to a demographic questionnaire and answered our language background survey. To ensure optimal communication for all participants, the testing team was composed of Deaf native signers as well as fluent English speakers. All Deaf participants were provided instruction in ASL, while all hearing participants were provided instructions in spoken English. The duration of the entire session was between an hour and an hour and a half, including set up. The project was approved by the University’s Institutional Review Board.

The task consisted of a delayed operation verification task of multiplication and subtraction problems. Problems could be single-digit small and large multiplication problems or single-digit small, large and two-digit extra-large subtraction problems (see list of problems in Appendix A). Extra-large problems were included to control for numerical magnitude and were created using the solution to large multiplication problems as minuend and one of the factors as the subtrahend. The delayed design was used to lock the ERP to the problem onset and model the ERP response for solving the operation rather than assessing answer plausibility or storage access.

Each problem was presented four times with the incorrect solution, twice per manipulation and four times with the correct solution. These were created based on prior literature and depending on the operation [26,37]. For multiplication problems, the incorrect solution was the answer to an adjacent problem in the same multiplication table. Each problem was presented twice with the solution to the problem right before and twice with the solution right after in the table. For example, 3 × 5 was presented twice with answer 10 (2 × 5) and twice with answer 20 (4 × 5). Additionally, the problem 5 × 3 was considered a distinct problem and presented with its own 2 incorrect solutions (12 and 18). For small and large subtraction problems, incorrect answers were created by adding or subtracting 1 or 2 from the correct solution, but for extra-large problems, they were created by adding or subtracting 2. Because subtraction problems rely on procedures, it was necessary to control for the parity of the proposed solution.

**Figure 1:**
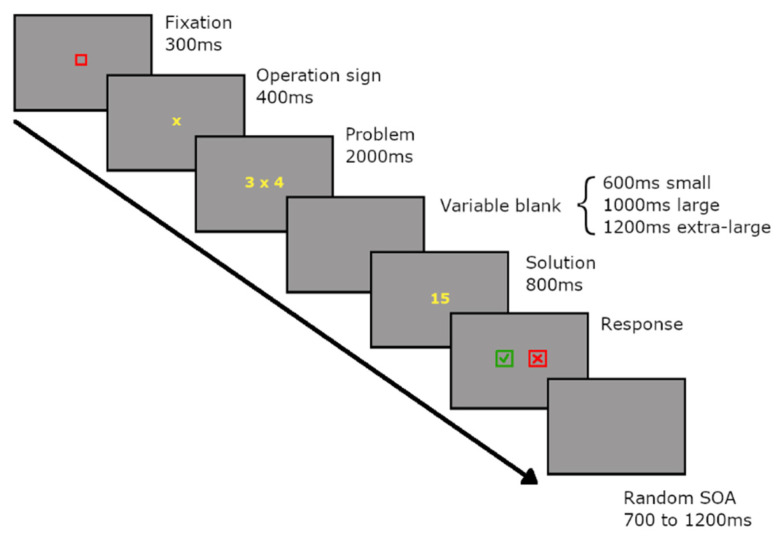
Schematic representation of a trial starting with a red fixation square for 300 ms, followed by a yellow multiplication or subtraction sign for 400 ms. The problem was then presented in yellow for 2000 ms, followed by a variable blank of 600 ms for small problems, 1000 ms for large problems and 1200 ms for extra-large problems. The solution was presented for 800 ms, and participants answered by selecting the green check or the red cross for correct or incorrect proposed solutions only on the next screen. The side of the presentation of the green check and red cross were counterbalanced and randomly presented. Participants would then press the f key or the j key on the keyboard ipsilateral to their selection. The response screen remained until participants gave their answer or for a maximum of 5000 ms.

In total, 480 problems (96 in each condition) were presented randomly in blocks of 48 trials (10 blocks). A trial would start with a red fixation box for 300 ms, then an operation sign (- or x) appeared for 400 ms before the problem was presented (Figure 1). This was done to avoid interference due to switching between operations [38,39]. The problem remained on the screen for 2000 ms and was replaced by a blank screen of variable duration. The duration was 600 ms for small problems, 1000 ms for large problems and 1200 ms for extra-large problems to provide participants with enough time to retrieve or calculate the solution. Next, a proposed solution was presented for 800 ms, for which participants only had to decide if it was accurate or not and hold their response to avoid motor components. On the following screen, a green check and red cross appeared until participants gave their responses. Participants pressed the key ipsilateral to the green check if they thought the proposed solution was accurate or by pressing the key ipsilateral to the red cross if they thought the answer was wrong. The side of appearance for the green check and red cross was counterbalanced and random to avoid motor preparation related to the response production. The trial was then followed by a blank screen with a random duration between 700 and 1200 ms (i.e., SOA).

### 2.3. Electroencephalography Data Acquisition and Preprocessing

The EEG was recorded using an actiCAP setup (Brain Products GmbH, Gilching, Germany) with 64 active Ag/AgCl electrodes and SuperVisc gel. The 64 channels were placed based on the 10/20 system onto caps of different sizes to fit the participant’s head optimally. EEG data were recorded at a rate of 1000 Hz with Cz as reference and AFz as ground with a low pass filter set at 280 Hz. Each electrode had an active amplifier and was connected to a 24-bit actiCHAMP amplifier for signal amplification (Brain Vision LLC, Morrisville, NC, USA). The impedance of all electrodes was kept at or below 50 k.

Preprocessing was performed using EEGLAB v2021.0 (https://eeglab.org/) (accessed on 15 June 2021) and the following toolboxes: PerpPipeline v0.55.4, Clean_rawdata v2.3, ICLabel v.1.3, and ERPLAB v8.10. Individual recordings were resampled to 250 Hz. A high-pass 0.5Hz filter at −6 dB with a transition bandwidth of 0.5 Hz (i.e., passband edge 0.25–0.75 Hz) was applied. An initial data cleaning was computed with PrepPipeline (v0.55.4) to remove line noise. Clean-rawdata (v2.3) with conservative parameters was used to reject bad channels and correct continuous data using Artifact Subspace Reconstruction (ASR). This was done to allow for subsequent optimal independent component analysis (ICA). Removed channels were then interpolated before the average re-referencing. Finally, the ICA was performed to remove eye blinks. These were identified with ICLabel and thorough inspection of the activity power spectrum graph. On average, 2.41 (SD = 0.84) and 2.43 (SD = 1.12) components were identified as eye blinks and removed for the ASL native signers and English-speaking groups, respectively.

To identify ERP components and the processes related to solving the arithmetic problem, epochs were created based on problem onset. The epochs start −500 ms before the problem onset (t = 0) and continue until 2500 ms after. Residual artifacts were identified and epochs with a peak-to-peak difference beyond 150 µv were removed using a 200 ms moving window and a 100 ms step over the duration of the entire epoch. For each participant, trials were averaged for each condition for correct trials only to avoid error-related noise. On average, 365 (SD = 96) epochs over a total of 480 were retained for the ASL native signers and 371 (SD = 74) epochs for the English-speaking group (*p* > 0.1). The entire duration before stimulus onset (−500 ms) was used as the baseline.

### 2.4. ERP Components and Analyses

**Figure 2:**
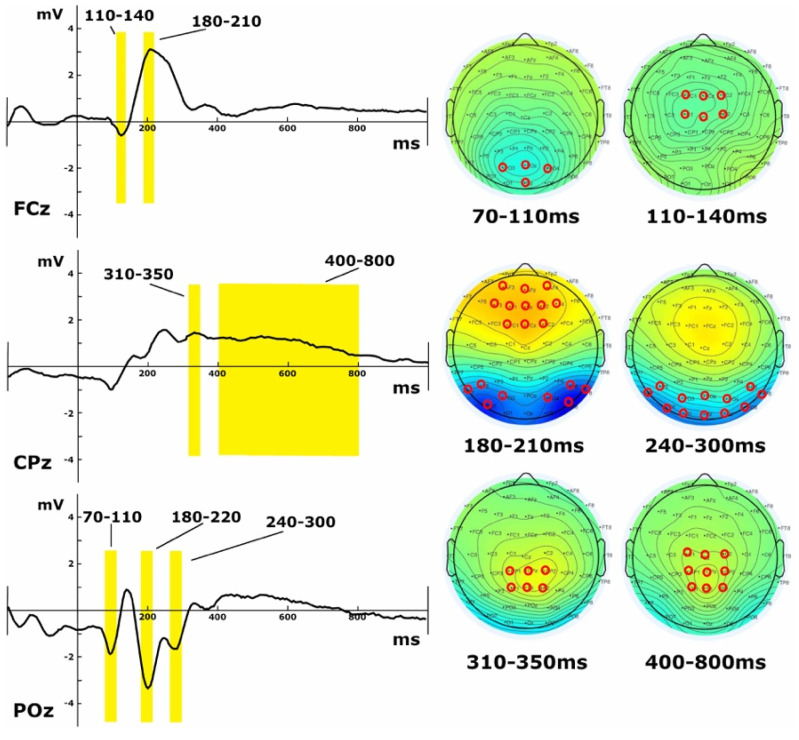
ERP and topographical representation of the seven components identified independently over the grand average waveforms computed across all tasks and both groups. On the left, the components are identified by yellow windows over representative channels: FCz, CPz and POz. On the right, the topographical maps represent average variations of the grand average across the scalp, with the ROIs identified by red rings.

To independently identify ERP components, time windows and regions of interest (ROIs) for further analyses, we created the grand average waveforms across all conditions and both groups. Observing the waveforms across the scalp and the topographical maps, we identified the following seven components (Figure 2): a first centro-posterior negativity between 70 ms and 110 ms over (O_3_, POz, PO_4_, Oz); a second negativity between 110 ms and 140 ms over the fronto-central electrodes (C_1_, Cz, C_2_, FC_1_, FCz, FC_2_); a first fronto-central positivity between 180 ms and 210 ms over (FC_1_, F_3_, F_1_, AF_3_), (FC_2_, F_4_, F_2_, AF_4_) and (FCz, Fz, AFz); at a similar timing, a bilateral parieto-occipital negativity between 180 ms and 220 ms over (PO_8_, PO_4_, P_8_, P_6_) and (PO_7_, PO_5_, P_7_, P_5_); a successive more central negativity encompassing the entire occipital and parietal channels between 240 ms and 300 ms over (O_1_, Oz, O_2_, PO_7_, PO_3_, POz, PO_4_, PO_8_, P_7_, P_5_, P_6_, P_8_); a centro-posterior positivity between 310 ms and 350 ms over (CP_1_, CPz, CP_2_, P_1_, Pz, P_2_); and finally, a long and late positive-going component around 400 ms until 800 ms over the centro-posterior channels (P_1_, Pz, P_2_, CP_1_, CPz, CP_2_, C_1_, Cz, C_2_). Time windows were created to ensure that the peaks for each channel comprising the ROI were within the time window while also avoiding the peaks of successive components.

Average amplitudes for each condition and each participant were then extracted and analyzed with repeated measures ANOVAs. Operation type (subtraction vs. multiplication) and problem size (small vs. large) were entered as within-subject variables and group (ASL signers vs. English speakers) was entered as a between-subjects variable. For components showing bilateral peaks or asymmetric topographies, laterality (left, center and right or left and right) was also entered as a within-subject factor. To further test whether specific components are related to problem size, a second set of repeated measures ANOVAs was performed entering the three subtraction sizes (i.e., small, large, and extra-large) as within-subjects and group as between-subjects. Finally, in instances where results needed further investigation to disentangle between competing explanations, further analyses were performed and specified where appropriate. For all ANOVAS, the significance threshold was set at α  = 0.05. When the assumption of sphericity was not met, the Huyn–Feldt correction was applied and the adjusted degrees of freedom were reported. We used the Benjamini–Hochberg False Discovery Rate [40] method to correct for multiple comparisons considering all planned ANOVAS over all components. To further investigate the interactions, the levels of the variables were tested with paired *t*-tests and the α was corrected using the Bonferroni method.

## 3. Results

### 3.1. Behavioral Results

**Figure 3:**
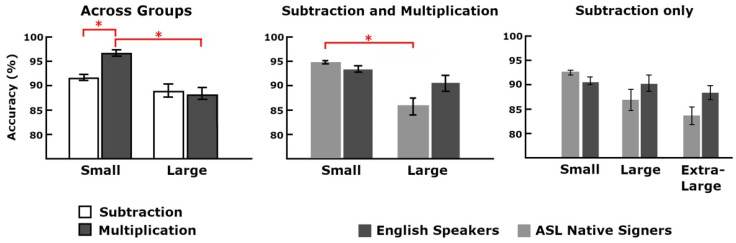
Graphs show percent accuracies for the operation type by problem size interaction on the left, group by problem size interaction merging both operation types in the center, and the problem size effect on the left for the three levels of subtraction. Between-group differences do not reach significance. The * denotes significant post-hoc differences.

Due to the experimental design, only task accuracy was analyzed. We first ran a repeated measures ANOVA with operation type and problem size as within-subject variables and group as between-subjects variables. The main effects of operation type (*F*(1, 62) = 17.61, *p* < 0.001, η^2^_p_ = 0.22) and problem size (*F*(1, 62) = 42.78, *p* < 0.001, η^2^_p_ = 0.41) were found to be significant. Responses to multiplication problems were more accurate than subtraction problems (μ = 94.5% and SD = 5.5% for multiplication vs. *M* = 90.3% and SD = 6.7% for subtraction) and responses to small problems were more accurate than large problems (*M* = 94.1% and SD = 3.3% for small vs. *M* = 88.6% and SD = 9.3% for large). The interactions operation type by problem size (*F*(1, 62) = 29.97, *p* < 0.001, η^2^_p_ = 0.33) and problem size by group (*F*(1, 62) = 11.53, *p* = 0.001, η^2^_p_ = 0.16) were also found to be significant (Figure 3). Post-hoc analyses, breaking down the operation type by problem size interaction, indicated that responses to small multiplication problems were significantly more accurate than large multiplication problems (*F*(1, 63) = 74.90, *p* < 0.001, η^2^_p_ = 0.54; 96.6% vs. 88.4%) and more accurate than small subtraction problems (*F*(1, 63) = 112.87, *p* < 0.001, η^2^_p_ = 0.64; 96.6% vs. 91.7%). The difference between small subtraction problems and large subtraction problems failed to reach significance (corrected α = 0.0125, *p* = 0.021; 91.7% vs. 88.9%) as well as for large subtraction and large multiplication problems (*p* = 0.56; 88.9% vs. 88.4%). Breaking down the problem size by group interaction, no group differences survived the multiple comparison (for small problems *p* > 0.05 and for large problems *p* = 0.045). Problem size, analyzed separately for each group, was significant only for ASL signers with responses to small problems being more accurate than large ones (*F*(1, 28) = 36.28, *p* < 0.001, η^2^_p_ = 0.56; 94.9% vs. 86.1% for ASL signers; *p* = 0.013; 93.5% vs.90.7% for English speakers).

We further tested with a repeated-measures ANOVA the three levels of the subtraction problems. Problem size was entered as a within-subject variable and group as a between-subjects. Only problem size survived multiple comparison correction, showing a linear decrease in accuracy with increasing problem size (*F*(1.9, 117.8) = 15.48, *p* < 0.001, η^2^_p_ = 0.20; 91.7%, 88.9%, and 86.3% from smallest to largest condition). Accuracies from small to extra-large for ASL signers were: 92.6%, 87.0%, and 83.7%; and for the English-speaking participants accuracies were: 90.9%, 90.5%, and 88.5%.

### 3.2. ERP Problem-Locked Components Results

#### 3.2.1. Centro-Posterior Negativity between 70 ms and 110 ms

The repeated measures ANOVA with operation type and problem size as within-subject factors and group as between-subjects variable revealed a significant effect of operation type (*F*(1, 62) = 16.56, *p* < 0.001, η*^2^*_p_ = 0.21) and problem size (*F*(1, 62) = 14.99, *p* < 0.001, η^2^_p_ = 0.19) but not group, nor any interaction. Subtraction problems and large problems yielded greater negativity at this stage (subtraction problems: −1.42 μV, multiplication problems: −1.05 μV, large problems −1.37 μV and small problems: −1.1 μV).

A second repeated measures ANOVA with problem size (small, large and extra-large) as within-subjects and group as between-subjects variables was also run for subtraction problems. Only the main effect of problem size was found significant (*F*(2, 124) = 5.51, *p* = 0.005, η^2^_p_ = 0.08; −1.25 μV, −1.58 μV and −1.34 μV for small, large and extra-large, respectively). Large subtraction problems were significantly more negative compared to both small and extra-large problems (corrected α = 0.017 for paired *t*-tests: *t*(63) = 3.07, *p* = 0.003 for small vs. large; *t*(63) = −2.46, *p* = 0.017 for large vs. extra-large). No difference was found between small and extra-large problems (*p* > 0.1)

#### 3.2.2. Fronto-Central Negativity between 110 ms and 140 ms

For the operation type by problem size by group analysis, we only found the main effect of group where ASL signers showed greater negativity compared to English-speaking participants (*F*(1, 62) = 9.83, *p* = 0.003, η^2^_p_ = 0.14; −0.77 μV vs. −0.05 μV for ASL signers and English speakers, respectively).

The repeated measure ANOVA comparing the three subtraction problem sizes again revealed a group effect (*F*(1, 62) = 9.64, *p* = 0.003, η^2^_p_ = 0.14; −0.80 μV vs. −0.01 μV for ASL signers and English speakers, respectively).

#### 3.2.3. Fronto-Central Positivity between 180 ms and 210 ms

For this component, the peak appeared skewed to the left; therefore, laterality (left, center and right) was added to the other three variables (operation type, problem size and group). Group was the only between-subjects variable. Laterality showed a significant result (*F*(1.49, 92.95) = 7.9, *p* = 0.002, η^2^_p_ = 0.11; 2.55 μV left, 2.63 μV center, and 2.31 μV right) with post-hoc paired *t*-test showing that the right channels were less positive compared to the central ones (corrected α = 0.017 for paired *t*-tests: *t*(63) = 4.72, *p* < 0.001 for center vs. right; *t*(63) = 2.28, *p* = 0.026 for left vs. right; *p* > 0.1 for left vs. center). Operation type was also significant with greater positivity for subtraction problems (*F*(1, 62) = 9.2, *p* = 0.004, η^2^_p_ = 0.13; 2.61 μV for subtraction and 2.38 μV for multiplication). No other effect was found.

The repeated measure ANOVA for the three subtraction sizes, laterality and group, only returned a significant effect of laterality (*F*(1.66, 103.11) = 7.81, *p* = 0.001, η^2^_p_ = 0.11, 2.6 μV for left, 2.72 μV for center and 2.38 μV for right). Post-hoc tests showed a significant difference for the right channels being less positive than the center ones (corrected α = 0.017 for paired *t*-tests: *t*(63) = 4.66, *p* < 0.001 for center vs. right; *t*(63) = 2.05, *p* = 0.044 for left vs. right; *p* > 0.1 for left vs. center).

#### 3.2.4. Bilateral Parieto-Occipital Negativity between 180 ms and 220 ms

This component appeared to show bilateral peaks; therefore, laterality with left and right was entered with the other variables into the repeated measures ANOVA. Right channels were more negative than left channels (*F*(1, 62) = 7.09, *p* = 0.01, η^2^_p_ = 0.10; −4.03 μV and −4.68 μV). No other effects were significant.

The repeated measures ANOVA for the three subtraction problem sizes also included laterality (left and right) in addition to group. Again, laterality was significant (*F*(1, 62) = 7.93, *p* = 0.006, η^2^_p_ = 0.11; −4.03 μV and −4.68 μV for right and left channels, respectively) as well as problem size (*F*(2, 124) = 5.12, *p* = 0.007, η^2^_p_ = 0.08) with small problems averaging −4.47 μV, large problems −4.48 μV, and extra-large problems −4.22 μV. Post-hoc paired *t*-test revealed that extra-large problems were less negative than the two other problem sizes (corrected α = 0.0167 for paired *t*-tests: *t*(63) = 2.49, *p* = 0.015 for small vs. extra-large; *t*(63) = 3.21, *p* = 0.002 for large vs. extra-large; *p* > 0.01 for small vs. large problems).

#### 3.2.5. Second Parieto-Occipital Negativity between 240 and 300 ms

The repeated measures ANOVA included only operation type, problem size and group. The main effects of operation (*F*(1, 62) = 23.6, *p* < 0.001, η^2^_p_ = 0.28) and size (*F*(1, 62) = 13.54, *p* < 0.001, η^2^_p_ = 0.18) were significant with multiplication problems being more negative than subtraction problems (−2.8 μV and −2.34 μV) and with small problems being more negative than larger ones (−2.67 μV and −2.46 μV). The main effect of group was not significant.

**Figure 4:**
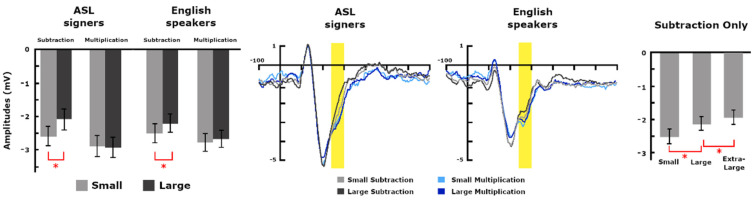
Modulation of amplitudes over the second posterior-occipital negativity between 240 ms and 300 ms. On the left, bars represent average amplitudes with standard errors for the two-way interaction, problem size by operation type, presented separately for each group for visualization purposes. In the center, the ERPs show average modulations over the parietal-occipital ROI for the time window between 240 ms and 300 ms (yellow highlight) for each group separately. On the right, the bars represent average modulations and standard errors for the three levels of the subtraction problems. Only subtraction problems show modulation with problem size over this component. The * denotes significant post-hoc differences.

The operation type by problem size interaction was also significant (*F*(1, 62) = 13.12, *p* = 0.001, η^2^_p_ = 0.18). Subtraction problems showed modulation for problem size with smaller problems being more negative than large problems (*F*(1, 63) = 20.21, *p* < 0.001, η^2^_p_ = 0.24; −2.53 μV and −2.15 μV). Multiplication problems did not show a significant modulation for size (*p* > 0.1). Both small and large problems were significantly more negative for multiplication than subtraction problems (*F*(1, 63) = 8.63, *p* = 0.005, η^2^_p_ = 0.12; −2.53 μV and −2.82 μV for small subtraction and small multiplication, respectively; and *F*(1, 63) = 27.83, *p* < 0.001, η^2^_p_ = 0.30; −2.15 μV and −2.78 μV for large subtraction and large multiplication, respectively). Importantly, there were no interactions with group (Figure 4).

Analyzing problem size for subtraction problems, we only found a main effect of size with a linear increase in amplitudes with increasing numerical size (*F*(2, 124) = 22.09, *p* < 0.001, η^2^_p_ = 0.26, with a significant linear trend *F*(1, 62) = 35.28, *p* < 0.001; average amplitudes of −2.53 μV for small, −2.15 μV for large and −1.96 μV for extra-large; post-hoc paired *t*-test comparisons with corrected α = 0.025: small vs. large *p* < 0.001, large vs. extra-large only close to significance with *p* = 0.03). No other effects were found. 

To test that the modulation of this component was not related to the numerical magnitude of the problems itself but depended on the cognitive process involved based on the operation, we directly compared large multiplication problems with extra-large subtraction problems as these were equated on overall magnitude. The repeated measure ANOVA with operation type as within-subject and group as between-subjects returned a significant operation type effect (*F*(1, 62) = 43.66, *p* < 0.001, η^2^_p_ = 0.41) with extra-large subtraction problems being significantly less negative than multiplication problem (−1.96 μV and −2.78 μV for extra-large subtraction and large multiplication problems, respectively). The effect of group was again not significant. 

Because extra-large problems were composed of two-digits minus one-digit operations while large multiplication problems were all single-digit operations, we also tested the possibility that the difference was related to perceptual difference or magnitude of the operands/minuends/subtrahends. Therefore, we ran a repeated-measures ANOVA with small subtraction problems and small multiplication problems only. These are highly similar in numerical magnitude and are all single-digit operations, thus making them perceptually similar. The effect was significant (*F*(1, 63) = 8.63, *p* = 0.005, η^2^_p_ = 0.12) with small multiplication being significantly more negative than small subtraction problems: −2.82 μV and −2.53 μV, respectively.

#### 3.2.6. P300 Centro-Posterior Positivity between 310–350 ms

The repeated-measures ANOVA with operation type, problem size and group showed only a main effect of group with ASL signers showing greater positivity (*F*(1, 62) = 8.48, *p* = 0.005, η^2^_p_ = 0.12; 1.59 μV and 0.91 μV for ASL signers and English speakers, respectively).

Investigating the three subtraction problem sizes, the repeated measures ANOVA again only returned a significant group effect with ASL signers showing more positive amplitudes (*F*(1, 62) = 7.37, *p* = 0.009, η^2^_p_ = 0.11; 1.61 μV and 0.98 μV for ASL signers and English speakers, respectively).

#### 3.2.7. LPC Late Centro-Posterior Positivity between 400 ms and 800 ms

Finally, the analysis over the late, positive-going component only returned the main effects of problem size (*F*(1, 62) = 7.38, *p* = 0.009, η^2^_p_ = 0.11). Small problems overall had more positive amplitudes (0.83 μV and 0.73 μV for small and large problems).

The repeated measures ANOVA on the three levels of the subtraction problems returned a significant group effect with greater positivity for ASL signers (*F*(1, 62) = 7.24, *p* = 0.009, η^2^_p_ = 0.11; 1.01 μV and 0.60 μV).

## 4. Discussion

In this experiment, we investigated whether a lifelong experience using a signed language would alter the neurocognitive processes involved in solving single-digit arithmetic problems. To do so, we compared Deaf native ASL signers and English-speaking participants using the ERP approach. Based on prior literature, we tested the two operations known for being most distinct within the hearing and speaking population: subtraction and multiplication problems [21,22]. These have shown to be solved through different procedures [41], rely on distinct brain networks [26,28] and modulate ERP components differently [23,25]. So far, one study investigated language modality and arithmetic processing using the fMRI [42]. Our study is unique as it is the first to investigate the impact of language modality on the time course of arithmetic processing. It is only assumed that the current models are independent of language modality and that native sign language users rely on the same cognitive processes as observed with hearing-speaking participants.

Behaviorally, the two groups performed overall equally well, and the main effects of problem size and operation type were found. Performance was overall highest for small multiplication problems, but no significant difference was observed for large problems across operation types. Deaf native signers showed a stronger problem size effect across both operations. Moreover, both groups showed a linear decrease in performance with increasing size for subtraction problems. Overall, both groups show similar behavioral effects for problem size and operation type.

For the analysis of the neural signatures, we calculated the ERPs in response to the presentation of the problem and independently identified seven components. The first four components appear within the 200 ms post-problem onset and are likely related to early visual and attentional processes [43], but that is also the time window where different ERP studies have shown that strategy selection can already occur (large two-digit problems [44]; split effects [45,46,47,48]). The remaining three may be considered later components and more susceptible to numerical quantity processing [32,33,37,49].

The first centro-posterior negativity between 70 and 110 ms shows modulation that is consistent with the visuospatial properties of the stimuli. Indeed, amplitudes are more negative overall for subtraction problems compared to multiplication problems and this could be due to large subtraction problems being composed of two digits minus one digit. A pattern suggesting activations related to the visual properties also appears in the analysis on the three levels of the subtraction problems. Although the patterns are not completely consistent with the variations in the number of digits in the subtraction problems, patterns are also not related to any magnitude variation within the problems. Importantly, they do not show any interaction suggesting early different processes occurring between the two groups. Given the very early component, the patterns are more likely explained by visuoperceptual variations in the stimuli.

For the second fronto-central negativity between 110 and 140 ms, we only find a group effect where ASL signers show greater negativity compared to English-speaking participants. Although we did not predict this result, studies on visual processing with congenitally deaf participants [50,51] have shown modulation of the visual-evoked potentials with greater negativity in the deaf group compared to the hearing group within this time window. This early modulation is interpreted as plasticity changes increasing the reliance on visual input. Our Deaf participants reported early, severe to profound hearing loss, which is consistent with prior results suggesting that a lack of early auditory input might have provided greater visuo-attentional resources.

Next, we found a fronto-central positivity between 180 and 210 ms, showing a lateralized modulation by operation. The left and central ROI channels were more positive than the right channels, and subtraction problems were more positive compared to multiplication problems. The absence of a size effect suggests that this component is not sensitive to the numerical magnitude but rather is related to the attentional and cognitive processes differently involved in the two operations. Regarding the operation type effect, it is known from prior literature that multiplication problems engage more left-lateralized processes compared to subtraction problems [23,26]. Zhou and colleagues [23], using ERPs, found lateralized effects for multiplication problems showing greater negativity over the frontal electrodes at around 320 ms. These effects were localized, through dipole source localization, in the left anterior brain and interpreted as greater reliance on phonological processes for multiplication problems compared to subtraction and addition problems. Consistent with these findings, we also find lower amplitudes for multiplication compared to subtraction problems; however, the effect appears over 100 ms earlier in our study. This timing difference could potentially be explained by differences in paradigm design. We intentionally presented the operation sign before the full problem to avoid trial-to-trial interference and cross-operation errors [38,52,53]. However, most importantly, behavioral work has shown that presenting the operation sign before the full problems decreases the response time for subtraction and addition problems selectively [41]. Although our paradigm does not allow to test for differences in response times, it is possible that priming the operation sign induced faster cognitive processing. Participants could have been ready to allocate resources differently by operation at an earlier time than in the study of Zhou and colleagues [23], where the full problem was presented at once. Interestingly, another study by Muluh et al. [36], which presented the full problem at once, also reported early operation differences on the P100 and P200 components post problem onset, thus as early as 100 ms to 150 ms. They interpret these early operation-related modulations as possible attention allocation differences, such as the orientation of brain resources, for encoding the operation signs and identifying the operation to be performed. Other studies using different paradigms than ours and specifically investigating strategy selection have found evidence for modulations related to differences in strategies already occurring in the first 200 ms window [44,45,46,47,48]. Therefore, given our paradigm, prior results and predictions, it is likely that our observed operation-related modulation around 200 ms is indicative of an early strategy selection that might favor phonological processing for multiplication problems. Most importantly to the present research question is that there was no modulation related to group at this early stage. This is the first evidence that the early differential processes involved for the two operations are likely to be similar regardless of the language modality used.

At a similar timing, but in the bilateral posterior areas, a negative component was observed between 180 ms and 220 ms. Interestingly, the topographical maps in Muluh and colleagues [36] show similar bilateral negativity between 200 ms and 250 ms and to be stronger for subtractions than multiplications, but the paper does not report any statistical data on this component. Our results showed that the right channels, subtraction problems, and ASL signers were overall more negative. Furthermore, investigating problem size within subtraction problems, we find again greater right-lateralized negativity as well as a problem size effect. The modulation with problem size is interesting as it does not appear to reflect a consistent modulation with either the visual properties of the problems or the numerical size. Extra-large problems were less negative than both small and large problems. A modulation based on size would predict a more gradual change in amplitudes for the three sizes. If this modulation were related to visual properties, both large and extra-large problems would show similar modulation given that both types of problems are two-digit minus two-digit operations. However, small and large subtraction problems, which differ on the number of digits, were not significantly different. This component could be, in part, showing complementary modulations to those observed simultaneously in the frontal positivity, that is operation and lateralization effects, as well as emerging components more clearly visible in the successive time window. Indeed, group and size effects appear to emerge and continue to modulate activations in the second posterior negativity.

In the second posterior negativity (Figure 4), between 240 ms and 300 ms, both operation type and problem size modulate the electrophysiological response. In support of the argument that the two consecutive posterior time windows indeed represent different components, we found that multiplication problems elicited greater negativity as opposed to subtraction problems being more negative in the prior time window. Small problems however continued to remain more negative than larger ones. Additionally, modulations with problem size differed based on operation type: the interaction was driven by a significant size modulation within subtraction problems only. For both groups, problem size was not significant for multiplication problems. This result supports prior findings that the two operations are solved through different processes [26]: Multiplication problems failed to show a modulation with problem size and, as shown in prior studies, this is consistent with the rote memorization hypothesis; The modulation with problems size for subtraction problems confirms the use of procedural and quantity strategies. The analysis including the three problem sizes for subtraction problems further confirms that the component is modulated by the numerical size of the problem as amplitudes showed a similar linear increase with increasing problem size across groups (Figure 4). Hence, this component is most likely induced by processes that relate to quantity manipulation.

Further analyses excluded that the modulation was related to overall numerical magnitude given that large multiplication problems were significantly more negative than extra-large subtraction problems (equated on numerical magnitude) and going in the opposite direction of the problem size modulation observed for subtraction problems. Importantly, behavioral analyses do not show any significant differences in performance between these two conditions excluding differences in the amount of attention or levels of performance. The ERP difference again supports the idea that distinct cognitive processes are occurring for the two operation types. Indeed, the extra-large subtraction problems used here are unlikely to be retrieved from long-term memory and thus required quantity manipulation. Further, we also excluded that the difference observed was related to the number of digits presented by comparing single-digit small problems across operation types. Despite being equated for the number of digits and numerical magnitude of the digits presented, amplitudes were significantly different. Again, this confirms the difference in strategies used for the two problems. Finally, and again most relevant to the current study, we did not observe any differences in amplitudes between groups for this component. It appears that the differential recruitment of strategies for the two types of problems holds similarly across the two groups.

Over the next component, the centro-posterior positivity between 310 and 350, there was only a main difference of group in both across and within operation analyses. ASL signers showed an overall greater positivity. The P300 is usually thought of as an indication of attentional demands, cognitive ability but also to be modulated by memory load. Studies on memory have shown that decreases in amplitudes were seen with increases in memory load [54]. Although the two groups do not show differences in how their brain responses differently modulate with operation type in the preceding stage, it is still possible that they rely differently on attentional and memory processes at later stages. Retrieving and manipulating linguistic information in sign language might recruit additional or different networks impacting this later component [55]. Indeed, studies on the neural correlates of sign language processes have shown both similar left-lateralized language networks but also additional right and parietal activations [18,55].

Finally, the late component covering the large window from 400 ms to 800 ms showed a size effect and a group effect. This late component has been reported previously to be present specifically for problems requiring greater procedural strategies. Núñez-Peña and colleagues [30,32] named this component the arithmetic-related positivity. In their results, this component was stronger for subtraction problems compared to addition problems [32] and for larger problems compared to smaller ones [30]. In our data, we do not find any operation differences, but we do find modulations driven by problem size when both operation types are merged. The direction of the modulation does not appear to be in line with prior findings. In our data, small problems, those more likely to be retrieved and less likely to be solved through procedural strategies, had more positive amplitudes. Comparing the three problem sizes for subtraction problems, we find a group difference with ASL-signers showing greater amplitudes but no problem size effect. The absence of the size effect suggests that this component in our study might not be related to quantity processing given our inclusion of extra-large problems certainly requiring quantity manipulation. Looking into studies on memory, specifically episodic memory, results have shown a positivity over parietal channels where greater amplitudes were related to greater retrieval success [56]. This could be more in line with our findings since smaller problems generally tend to rely more on memory retrieval. It is also possible that the two groups differ in terms of episodic memory reliance. Because the task resulted in several repetitions of the same problem, it is plausible that participants recollected answers from a prior calculation or prior retrieval. This could have been easier for problems that were more easily answered, such as smaller problems and, more speculatively, might be influenced by language modality. It may be that using sign language, compared to a spoken language, increases episodic memory encoding while doing the task. This remains an interesting open question.

Our results so far show that the two groups rely on similar distinct attentional and quantity processing mechanisms for solving subtraction and multiplication operations. These results further support the idea that the cognitive processes recruited while solving different arithmetic problems are independent of the language modality used and, most likely, from the language modality in which operations were learned. Based on the recycling hypothesis, humans are not born with predefined brain areas supporting higher-level arithmetical thinking [57]. It is through the recycling of older core systems that the human mind can reach symbolic thought and mathematical reasoning. What we observe here is that even through very different prolonged language and sensory experiences, the distinction between the operations remains, and the attentional and cognitive processes involved are surprisingly similar across groups. Subtle educational and short training manipulations have shown changes in brain networks and strategies [3,4]; how is it that the use of a visual and manual language does not modify more extensively the processes involved? One possible reason might be that language is foundational to abstract thought and that the modality is processed early in the cognitive stream and then filtered out as to leave only abstract reasoning at play. Current research brings evidence for both function-specificity in some of the key language brain areas and language modality-specific effects. Indeed, the left-lateralized language network has repeatedly been shown to be activated regardless of language modality and supporting similar cognitive processes across modalities [16,17,18,58,59,60,61,62]. This supports the idea that core language processes are subtended similarly and in similar brain regions regardless of modality. However, there is also evidence for language modality-specific activations such as greater bilateral network recruitment with additional spatial-related processes in the parietal lobes for sign language processing. Morford et al. [63] found modality-specific interference effects, suggesting that the modality is not completely filtered out early in the decoding process. The other possibility is that, regardless of the language modality and its processing, something in the characteristics of the operation themselves dictate how the core systems are being recruited to support proficiency.

Because this is the first ERP study investigating arithmetic in Deaf native signers, we acknowledge that much is still to be investigated. Even if we do not evidence differences between groups in our paradigm using visual input and Arabic digits, we acknowledge that the question of the impact of language modality is still open. Indeed, more studies investigating more subtly the presentation modality could inform on the role of language modality as well as the abstractness of arithmetic processing. For example, it would be relevant to investigate how presenting arithmetic problems in the language modality of the participants (i.e., in spoken vs. signed language) might modulate the cognitive processes recruited.

Our findings are also relevant to the Deaf population and the education of deaf children. Visual-signed languages are still not fully recognized around the world, and many deaf children are still withheld from full language access based on preconceptions about sign languages. Here, we selected highly proficient ASL signers with profound to severe hearing loss, who reported being exposed to ASL prior to age two and having received instruction in ASL through their formative years. However, their behavioral and neural profile appears exceptionally in line with that presented by our hearing group. These results, along with those on ASL language processing, once more dispel the belief that exposure to a sign language should be withheld. On the contrary, these results support the idea that the brain does not care about language modality, provided it is given optimal access to language. We find that the attentional processes and the differentiation in the neural recruitment for arithmetic processing appears to be immune to language modality. We hope that this is only the first of many studies further investigating the role of language modality on the neurocognitive processes supporting arithmetic.

## 5. Conclusions

In summary, to answer the question of whether Deaf native signers and English-speakers process arithmetic operations similarly, we find evidence for similar distinctions between operations for the two groups. Further, the pattern of modulations with problem size is also similar across groups. Indeed, in both groups, there was an early operation-dependent frontal modulation and a posterior size-dependent modulation only for subtraction problems, suggesting a similar operation-specific quantity modulation. Our results therefore bring no evidence indicating that the two linguistic groups resort to substantially different strategies and that using a sign language impacts the cognitive processes recruited in solving arithmetic operations.

## Data Availability

The data presented in this study are available on request from the corresponding author.

## Appendix A

**Table A1:** List of arithmetic problems, their correct solutions and the two alternative incorrect solutions used as stimuli. Details on item selection and construction are provided in the methods sections.

Operation	Size	1st Operand (x)	2nd Operand (y)	Correct Solution (z)	Incorrect Smaller	Incorrect Larger
Subtraction	Small	5	3	2	1 (z − 1)	3 (z + 1)
Subtraction	Small	6	4	2	1 (z − 1)	3 (z + 1)
Subtraction	Small	7	5	2	1 (z − 1)	3 (z + 1)
Subtraction	Small	7	4	3	2 (z − 1)	4 (z + 1)
Subtraction	Small	8	5	3	2 (z − 1)	4 (z + 1)
Subtraction	Small	9	5	4	3 (z − 1)	5 (z + 1)
Subtraction	Small	5	2	3	1 (z − 2)	5 (z + 2)
Subtraction	Small	6	2	4	2 (z − 2)	6 (z + 2)
Subtraction	Small	7	2	5	3 (z − 2)	7 (z + 2)
Subtraction	Small	7	3	4	2 (z − 2)	6 (z + 2)
Subtraction	Small	8	3	5	3 (z − 2)	7 (z + 2)
Subtraction	Small	9	4	5	3 (z − 2)	7 (z + 2)
Subtraction	Large	13	7	6	5 (z − 1)	7 (z + 1)
Subtraction	Large	14	8	6	5 (z − 1)	7 (z + 1)
Subtraction	Large	15	9	6	5 (z − 1)	7 (z + 1)
Subtraction	Large	15	8	7	6 (z − 1)	8 (z + 1)
Subtraction	Large	16	9	7	6 (z − 1)	8 (z + 1)
Subtraction	Large	17	9	8	7 (z − 1)	9 (z + 1)
Subtraction	Large	13	6	7	5 (z − 2)	9 (z + 2)
Subtraction	Large	14	6	8	6 (z − 2)	10 (z + 2)
Subtraction	Large	15	6	9	7 (z − 2)	11 (z + 2)
Subtraction	Large	15	7	8	6 (z − 2)	10 (z + 2)
Subtraction	Large	16	7	9	7 (z − 2)	11 (z + 2)
Subtraction	Large	17	8	9	7 (z − 2)	11 (z + 2)
Subtraction	Extra-Large	42	6	36	34 (z − 2)	38 (z + 2)
Subtraction	Extra-Large	48	6	42	40 (z − 2)	44 (z + 2)
Subtraction	Extra-Large	54	6	48	46 (z − 2)	50 (z + 2)
Subtraction	Extra-Large	56	7	49	47 (z − 2)	51 (z + 2)
Subtraction	Extra-Large	63	7	56	54 (z − 2)	58 (z + 2)
Subtraction	Extra-Large	72	8	64	62 (z − 2)	66 (z + 2)
Subtraction	Extra-Large	42	7	35	33 (z − 2)	37 (z + 2)
Subtraction	Extra-Large	48	8	40	38 (z − 2)	42 (z + 2)
Subtraction	Extra-Large	54	9	45	43 (z − 2)	47 (z + 2)
Subtraction	Extra-Large	56	8	48	46 (z − 2)	50 (z + 2)
Subtraction	Extra-Large	63	9	54	52 (z − 2)	56 (z + 2)
Subtraction	Extra-Large	72	9	63	61 (z − 2)	65 (z + 2)
Multiplication	Small	2	3	6	3 (x − 1)	9 (x + 1)
Multiplication	Small	2	4	8	4 (x − 1)	12 (x + 1)
Multiplication	Small	2	5	10	5 (x − 1)	15 (x + 1)
Multiplication	Small	3	4	12	8 (x − 1)	16 (x + 1)
Multiplication	Small	3	5	15	10 (x − 1)	20 (x + 1)
Multiplication	Small	4	5	2	15 (x − 1)	25 (x + 1)
Multiplication	Small	3	2	6	4 (x − 1)	8 (x + 1)
Multiplication	Small	4	2	8	6 (x − 1)	10 (x + 1)
Multiplication	Small	5	2	10	8 (x − 1)	12 (x + 1)
Multiplication	Small	4	3	12	9 (x − 1)	15 (x + 1)
Multiplication	Small	5	3	15	12 (x − 1)	18 (x + 1)
Multiplication	Small	5	4	20	16 (x − 1)	24 (x + 1)
Multiplication	Large	6	7	42	35 (x − 1)	49 (x + 1)
Multiplication	Large	6	8	48	40 (x − 1)	56 (x + 1)
Multiplication	Large	6	9	54	45 (x − 1)	63 (x + 1)
Multiplication	Large	7	8	56	48 (x − 1)	64 (x + 1)
Multiplication	Large	7	9	63	54 (x − 1)	72 (x + 1)
Multiplication	Large	8	9	72	63 (x − 1)	81 (x + 1)
Multiplication	Large	7	6	42	36 (x − 1)	48 (x + 1)
Multiplication	Large	8	6	48	42 (x − 1)	54 (x + 1)
Multiplication	Large	9	6	54	48 (x − 1)	60 (x + 1)
Multiplication	Large	8	7	56	49 (x − 1)	63 (x + 1)
Multiplication	Large	9	7	63	56 (x − 1)	70 (x + 1)
Multiplication	Large	9	8	72	64 (x − 1)	80 (x + 1)
